# Pathogens that infect mammalian cells via sulfonated glycosaminoglycans

**DOI:** 10.3389/fcimb.2025.1613923

**Published:** 2025-06-10

**Authors:** Jessica S. Morris, Paul A. Dawson

**Affiliations:** Mater Research Institute - University of Queensland, Translational Research Institute, Woolloongabba, QLD, Australia

**Keywords:** sulfate, virus, bacteria, parasite, infection, glycosaminoglycan, proteoglycan

## Abstract

Sulfonated glycosaminoglycans, such as heparan sulfate and dermatan sulfate, form major components of the cell surface and extracellular matrix, and display vital roles in mammalian physiology, including growth and development. The identification of specific binding to different glycosaminoglycans by a variety of pathogens has led to increased interest in this mechanism for understanding infection. Over the past four decades there have been more than 300 studies on various pathogens that utilize glycosaminoglycans in their infection process. Currently, no articles have collated all known pathogens that use this process. So it is timely that this article provides an overview of all known pathogens that use glycosaminoglycans to enhance their binding and/or infection in human cells. This was done by using the search terms “sulfate/sulphate” “pathogen”, “virus”, “bacteria”, “parasite”, “infection” and “glycosaminoglycans” to curate peer-reviewed and relevant original research articles from PubMed. This search found that glycosaminoglycans are used in the infection process for 59 viruses, 28 bacteria, and 8 other pathogens (i.e. parasitic protozoa, prions). These findings highlight the conserved and widespread use of glycosaminoglycans for enhancing pathogen infection. In addition, the curated list of pathogens in this study provides a resource for future studies to consider potential therapeutic approaches for targeted disruption of the interaction between pathogens and glycosaminoglycans.

## 1 Introduction

**Figure 1:**
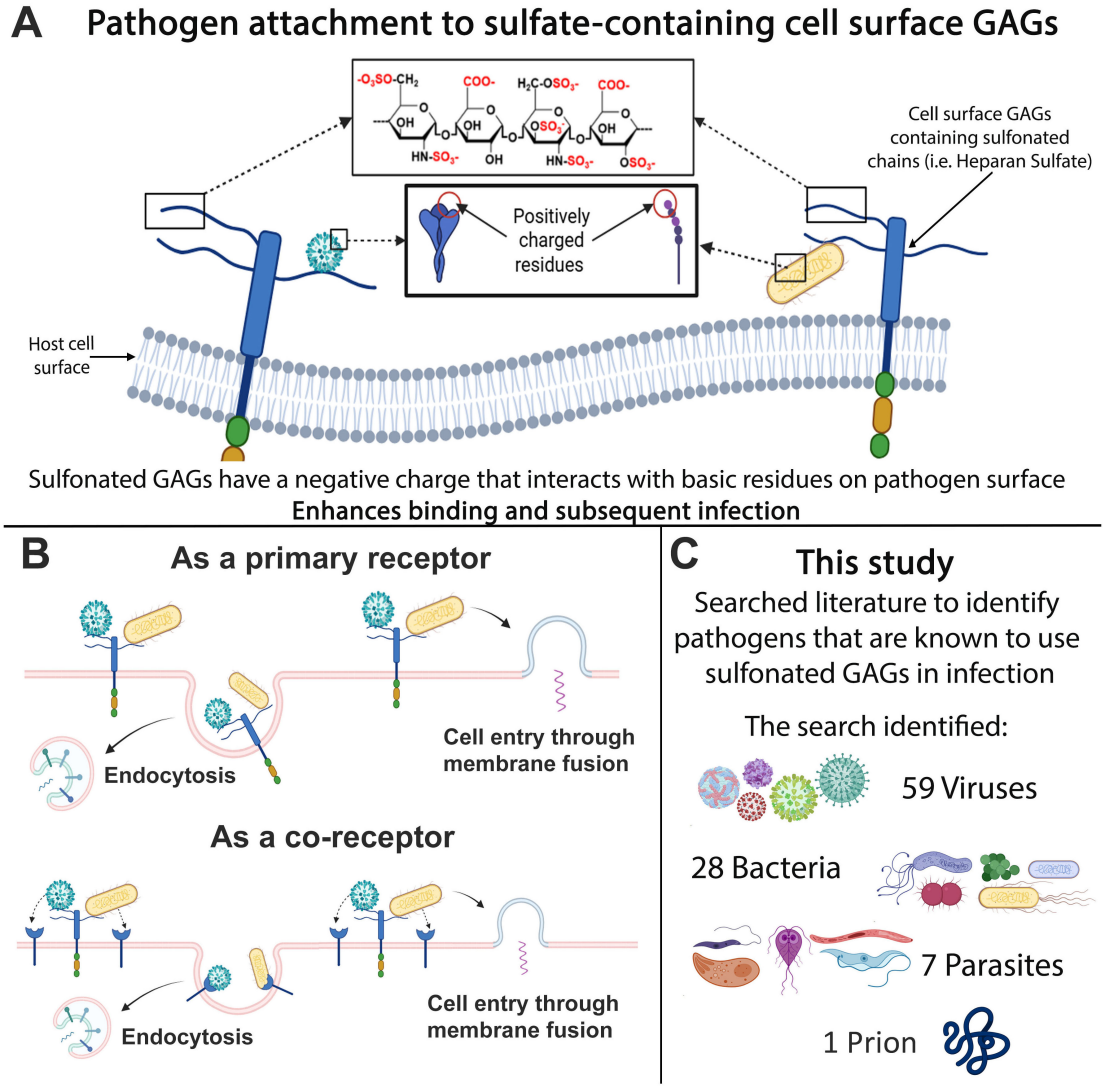
GAG-pathogen interactions. **(A)** Electrostatic interactions and **(B)** the functional roles of GAGs in pathogen binding and entry. **(C)** Summary of pathogens that infect mammalian cells via glycosaminoglycans.

Sulfate (SO_4_^2-^) plays a critical role in modulating numerous molecular and cellular functions in mammalian physiology (Dawson et al., 2015a). Conjugation of sulfate (sulfonation) to glycosaminoglycans (GAGs) plays an important role in maintaining the structure and function of tissues throughout the body. Several GAGs, including heparan sulfate (HS) and dermatan sulfate (DS), are major components of the cell surface and extracellular matrix (Wang and Chi, 2022). The attachment of numerous pathogens to mammalian host cells is enhanced by the sulfate content of GAGs. Sulfate provides a negative charge, leading to an electrostatic interaction with the basic residues of the pathogen surfaces that increases pathogen concentration at the host cell surface (**Figure 1A**), thus enhancing more efficient infection (Carvajal-Barriga and Fields, 2023; Lauster et al., 2023). Since the recent COVID pandemic, research into the role of sulfonated GAGs and enhanced pathogen infection has increased with the finding of HS as an attachment receptor for SARS-CoV-2 (De Pasquale et al., 2021).

Importantly, a sufficient supply of sulfate is needed to maintain the required sulfate content of GAGs (Cole and Evrovski, 2000; Dawson et al., 2003; Dawson et al., 2009). This is highly relevant when considering the requirement of GAGs for enhancing pathogen binding and entry. Circulating sulfate levels are altered by diet, pharmaceuticals, certain physiological conditions and genetics (Dawson, 2013). By inference, these factors which impact sulfate supply from circulation are proposed to subsequently compromise or enhance infection of GAG-binding pathogens.

Previous studies have focused predominantly on certain pathogens that are known to interact with GAGs. This study aimed to provide an overview of all viral, bacteria and parasitic pathogens that are known to interact with GAGs, leading to enhanced mammalian cell infection. This was done by using the search terms “sulfate/sulphate”, “pathogen”, “virus”, “bacteria”, “parasite”, “infection” and “glycosaminoglycans” to curate peer-reviewed research articles from PubMed, with searches done between February to November 2024. The articles returned from these searches were filtered for English, screened for duplicates and relevance and then reviewed to compile a list of pathogens. It was found that the use of GAGs is a highly conserved feature in the infection process for 95 pathogens (59 viruses, 28 bacteria, 7 parasites and 1 prion). These findings provide information for future studies of pathogen infection and those factors that increase or decrease the sulfate content of GAGs.

## 2 Sulfate biology

In humans, sulfate is obtained from diet and the intracellular catabolism of sulfur-containing amino acids (Dawson et al., 2015a). Dietary sulfate is absorbed via the intestinal epithelium and supplies approximately a third of daily sulfate requirements (Dawson, 2013). However, intake can vary greatly (1.5–16 mmol/day) depending on types of food consumed and source of drinking water (Dawson, 2013). Circulating sulfate levels are maintained by the kidneys, which filter sulfate in the glomerulus and then reabsorb sulfate in the proximal tubule (Dawson et al., 2015a).

Sulfate reabsorption is mediated by two sulfate transporters; SLC13A1 is located on the apical membrane where it mediates the first step of reabsorption, and SL26A1 which mediates the second step across the basolateral membrane (Karniski et al., 1998). Tissue-specific sulfate transporters mediate the uptake of sulfate from circulation into cells, which is then used to generate 3’-phosphoadenosine 5’-phosphosulfate (PAPS) by PAPS synthetase. The sulfonate group from PAPS is transferred via sulfotransferases to a wide range of endogenous and exogenous molecules (McCarver and Hines, 2002). Sulfate conjugation (sulfonation) alters the physiological properties of molecules including: (i) clearance and detoxification of xenobiotics and certain pharmaceutical drugs (McCarver and Hines, 2002); (ii) inactivation of neurotransmitters, steroids and thyroid hormone (McCarver and Hines, 2002; Dawson, 2012); and (iii) maintenance of tissue structure and function by altering sulfate content of GAGs (Sarrazin et al., 2011). Disturbances within any of these sulfate pathways, and subsequently the balance of sulfonated and unconjugated substrates, has the potential to modify the biophysical properties of cells.

## 3 Factors impacting circulating sulfate levels

In humans, circulating sulfate level is approximately 300 µmol/L but this can be altered by physiological, environmental and genetic factors (Cole and Evrovski, 2000). Diet is a significant contributing factor to sulfate levels, with food (~0.85 g SO_4_^2-^/day) and drinking water (~0.78 g SO_4_^2-^/day) accounting for approximately one third of estimated sulfate requirements (Allen et al., 1989; Florin et al., 1991; Florin et al., 1993). Animal studies have also shown that restricting dietary intake of sulfate intake can lead to hyposulfatemia and reduced sulfonation capacity, which can be reversed by sulfate supplementation (McGarry and Roe, 1973; Price and Jollow, 1989; Hou et al., 2003; Pecora et al., 2006). Additionally, ingestion of some phenolic-based pharmaceuticals that are metabolized by sulfonation are also known to decrease circulating sulfate levels (Kauffman, 2004).

In pregnancy, circulating sulfate concentrations increase significantly with levels peaking in late gestation (Dawson et al., 2015b). This increased sulfatemia is mediated by up-regulation of sulfate reabsorption due to a 2-fold increase in SLC13A1 expression in the maternal kidneys (Dawson et al., 2012; Dawson et al., 2015b). This provides a reservoir to meet the needs of the developing fetus, which has negligible capacity to generate sulfate until late gestation and thereby, is completely reliant on the maternal sulfate supply (Dawson, 2011).

Chronic kidney disease (CKD) is another physiological condition known to affect circulating sulfate levels, increasing by approximately 2-fold due to reduced glomerular filtration rate (Yildirim et al., 2019). Previous studies have shown a reduction in serum sulfate by more than 60% in CKD patients following 6 hours of dialysis (Freeman and Richards, 1979).

More than 90 genes are involved in the maintenance of sulfate homeostasis, including those encoding sulfate transporters (Langford et al., 2017). Previous studies have shown that targeted disruption of *Slc13a1* leads to hypersulfaturia, hyposulfatemia and reduced sulfonation capacity in mice (Dawson et al., 2003). Additionally, loss-of-function mutations in human *SLC13A1* gene that cause hypersulfaturia and hyposulfatemia have also been identified (Bowling et al., 2012; Tise et al., 2025). To date, 752 validated non-synonymous (ns) single nucleotide polymorphisms (SNPs) in *SLC13A1* have been identified, more than 400 of which are predicted to disrupt sulfate transport (Dawson and Markovich, 2007; Langford et al., 2017). *SLC13A1* has an uncommonly high ratio (Ka: Ks ≈4:1) of nsSNPs to synonymous SNPs, which is consistent with a strong positive selection for evolutionary change (Kreitman and Comeron, 1999; Dawson and Markovich, 2007). The high Ka: Ks ratio found in *SLC13A1*, together with the high allelic frequency (range = 22.5 to 40.4%) of N174S which leads to ≈60% loss of sulfate transport function (Lee S. et al., 2006), implies that reduced SLC13A1 function, and subsequent decrease in circulating sulfate level, may have provided a biological benefit to human evolution.

In conclusion, circulating sulfate levels are altered by diet, pharmaceuticals, certain physiological conditions and genetics (Dawson, 2013). Furthermore, low sulfate levels have been linked to a decrease in sulfonation capacity and sulfate content of resulting substrates, including cell-surface GAGs (Dawson et al., 2009). The negative charge conferred by sulfate is an important factor in cellular processes mediated by GAGs, such as the internalization of macromolecules, therefore a decrease in sulfonation capacity has the potential to disrupt these processes (Wadstrom and Ljungh, 1999).

## 4 Sulfonated glycosaminoglycans

All GAGs contain *O*-sulfonation, while heparan sulfate (HS) also contains *N*-sulfonation (Rudd et al., 2010). The degree of sulfonation and overall sulfate content of GAGs is dependent on circulating sulfate levels, which are impacted by various factors as described above. Sulfonation of various hydroxyl groups or amino groups present on the glucosamine component determines its ability to interact with various proteins and subsequently its bioactive function (Afratis et al., 2012).

HS consists of repeating disaccharide units of *N*-acetylglucosamine and hexuronic acid (Casale and Crane, 2025). HS is tethered to a proteoglycan (PG) core protein core via a serine residue connected to a tetrasaccharide (Casale and Crane, 2025). Chondroitin sulfate (CS) and dermatan sulfate are very similar in structural composition to HS, with the primary difference being the presence of *N*-sulfates present in HS (Rudd et al., 2010). Keratin sulfate (KS) consists of repeating galactose and *N*-acetylglucosamine disaccharides, with sulfation present on either unit of the disaccharide repeat. Unlike other GAGs, KS is not connected via a tetrasaccharide linker to the PG core. Instead, the three subtypes of KS (KSI, KSII and KSIII) each use a unique mechanism for linkage to the PG core. KSI GAG chains are tethered by a complex glycan structure utilizing an asparagine amino acid link, KSII chains have an *N*-acetylgalactosamine link via serine or threonine residues, and KSIII has a mannose linker via serine or threonine residues (Prydz, 2015). The molecular structure of individual GAGs determines their resulting properties, including their affinity for binding other molecules (Casale and Crane, 2025).

The negative charge of GAGs is known to enhance the binding and internalization of macromolecules, including various viral, bacterial and parasitic pathogens (Wadstrom and Ljungh, 1999; De Pasquale et al., 2021). Many viruses, including SARS-CoV 2 (Chu et al., 2021), Dengue virus (DENV) (Artpradit et al., 2013) and Herpes Simplex Virus (HSV) (O’Donnell and Shukla, 2008) bind to GAGs as a receptor for their initial attachment to host cells (**Figure 1B**). Several bacteria, such as *Listeria monocytogenes* (Henry-Stanley et al., 2003), *Mycobacterium tuberculosis* (Zimmermann et al., 2016) and *Pseudomonas aeruginosa* (Bucior et al., 2012), similarly utilize GAGs for attachment to host cells. Additionally, several bacterial pathogens induce the release of DS or HS from cell surface to counteract cationic antimicrobial factors or neutrophil-mediated host defense mechanisms (Park et al., 2001; Schmidtchen et al., 2001; Park et al., 2004; Chen et al., 2007). Furthermore, several pathogens have also been shown to subvert GAGs to prevent detection by immune mechanisms (Chen et al., 2008; Aquino and Park, 2016). Altogether, these studies suggest that GAG–pathogen interactions and subversion of GAG functions are important virulence mechanisms for a wide variety of pathogens.

While GAG-binding occurs in regions of positive charge within the binding proteins of pathogens, it is not simple to predict. Arginine residues are seen to bind more tightly to GAGs than lysine despite having identical net charges (Eilts et al., 2023). It has also been suggested that certain spacing between basic residues may be critical for binding to occur (Eilts et al., 2023). For some GAG-pathogen interactions, the degree and sequence of polymerization and sulfonation have been observed to impact binding affinity (Mitra et al., 2021). For example, CMV has been observed to preferentially bind HS with higher degrees of polymerization and sulfonation (Mitra et al., 2021).

This review brings together all known viruses, bacteria and parasites that utilize GAGs to bind and infect mammalian host cells. It also aims to curate information from those studies exploring the relationship between the sulfate content of GAGs and potential for infection. This knowledge provides a resource for future studies into the role of pathogen invasion into host cells via GAGs and how this may be impacted by those factors which are known to alter circulating sulfate level.

## 5 Pathogens that utilize sulfonated GAGs for infection

### 5.1 Viruses

This study identified that binding of GAGs for entry into mammalian cells is conserved across at least 6 virus families; alphaviridae, flaviviridae, coronaviridae, picornaviridae, orthoherpiviridae and paramyxoviridae. In total, 59 viruses were identified as interacting with GAGs for *in vivo* infection or shown to rapidly adapt to bind GAGs in cultured cell lines (**Table 1**).

**Table 1:** Viral pathogens that interact with sulfonated GAGs during infection process.

Family Virus	Mechanism	References
Alphaviridae		
Chikungunya	Binding HS essential for entry into host cell	(Gardner et al., 2012)
Sinbis Virus	Binding HS increases efficiency but not required for attachment	(Byrnes and Griffin, 2000)
Eastern Equine Encephalitis Virus	Binding HS increases efficiency but not required for attachment	(Gardner et al., 2011)
Venezuelan Equine Encephalitis virus	Rapidly adapts to bind HS in cell culture	(Bernard et al., 2000)
Ross River Virus	Binds HS as a coreceptor in some strains	(Heil et al., 2001; Zhang W. et al., 2005)
Semliki Forest Virus	Rapidly adapts to bind HS in cell culture	(Smit et al., 2002)
Flaviviridae		
Dengue Virus	Interacts with HS as an attachment factor. Secreted NS1 protein accumulates on infected cell membranes and interacts with HS and CS-E on cell surface, leading to selective vascular leak syndrome	(Lee E. et al., 2006; Artpradit et al., 2013; Wang and Chi, 2022)
Tick-Bourne Encephalitis Virus	Rapidly adapts to bind HS in cell culture, and when cultured in sulfate-deficient conditions growth of virus is delayed	(Mandl et al., 2001)
Japanese Encephalitis Virus	Binding HS and DS increases efficiency but not required for attachment and entry.	(Lee et al., 2004; Ling et al., 2022)
West Nile Virus	Binds HS as a cofactor. Although, increased GAG affinity is associated with decreased neuroinvasiveness	(Ling et al., 2022)
Yellow Fever Virus	Binds HS and infection is significantly reduced when HS is desulfonated or enzymatically removed from cell surface	(Germi et al., 2002)
Murray Valley Encephalitis	Binds HS as a cofactor. Although, increased GAG affinity is associated with decreased neuroinvasiveness	(Lee et al., 2004)
Hepatitis C	Binding HS essential for entry into host cell (6-O and N-sulfation required but not 2-O sulfation)	(Xu et al., 2015)
Zika Virus	Rapidly adapts to bind HS and other GAGs in cell culture. Sulfonation patterns observed to affect binding affinity	(Kim et al., 2017; Tan et al., 2017)
Conoronaviridae		
SARS-CoV	Binds HS as an attachment factor	(Lang et al., 2011)
SARS-CoV-2	Binding HS as a cofactor is essential for entry into host cell	(Clausen et al., 2020; De Pasquale et al., 2021)
HCoV-NL63	Binding HS as a cofactor is essential for entry into host cell	(Milewska et al., 2014)
MERS	Binding HS as a cofactor may be essential for entry into host cell	(Hao et al., 2021)
Herpesviruses		
Cytomegalovirus	Binding HS is essential for infection. Degree of polymerization and sulfation patterns in HS critical for entry into host cells	(Compton et al., 1993; Mitra et al., 2021)
Varicella zoster virus	Binding HS is essential for entry into host cell	(Zhu et al., 1995)
Hyman herpes virus 7	Binding HS increases efficiency but not required for attachment	(Skrincosky et al., 2000)
Kaposi's sarcoma-associated virus	Binding HS essential for entry into host cell	(Birkmann et al., 2001)
Epstein-Barr Virus	Binds HS but binding appears to be non-productive	(Chesnokova et al., 2016)
Herpes Simplex Virus	Binding HS is essential for entry into host cell	(Trybala et al., 2000; O’Donnell et al., 2010)
Picornaviridae		
Enterovirus 71	Binds HS as an attachment factor but not essential for entry	(Tseligka et al., 2018)
Coxsackievirus A9	Binds HS as an attachment factor- essential for some strains	(Merilahti et al., 2016)
Coxsackievirus A16	Binds HS as an attachment factor but not essential for entry	(Merilahti et al., 2016)
Coxsackievirus B3	Rapidly adapts to bind HS in cell culture	(Wang and Pfeiffer, 2016)
Rhinovirus 8	Binds HS to facilitate entry into host cell	(Khan et al., 2011)
Rhinovirus C15	Rapidly adapts to bind HS in cell culture	(Bochkov et al., 2016)
Rhinovirus 54	Binds HS as an attachment factor but not essential for entry	(Khan et al., 2007)
Rhinovirus 89	Rapidly adapts to bind HS as primary receptor in cell culture	(Vlasak et al., 2005)
Echovirus 5 (EV)	Binds HS as an attachment factor but not essential for entry	(Israelsson et al., 2010)
Echovirus 6 (EV)	Binds HS as an attachment factor but not essential for entry	(Goodfellow et al., 2001)
Human parechovirus 1	Binds HS as an attachment factor and may be essential for entry	(Merilahti et al., 2016)
Adenoviridae		
Adenovirus 3 and Adenovirus 5	Binds HS as a coreceptor for infection- likely operates to determine host tropism	(Dechecchi et al., 2001; Zaiss et al., 2016)
Paramyxoviridae		
Hendra virus	Binds HS as attachment factor in circulating leukocytes thereby promoting viral dissemination.	(Mathieu et al., 2015)
Nipah Virus	Use HS as attachment factor- specifically in circulating leukocytes thereby promoting viral dissemination	(Mathieu et al., 2015)
Respiratory Syncytial Virus	Binds HS as an attachment factor, may be essential for entry	(Donalisio et al., 2012; Johnson et al., 2015)
Parainfluenza virus 3	Binds HS to facilitate entry into host cell	(Bose and Banerjee, 2002; Zhang L. et al., 2005)
Human Metapneumovirus	Binds HS as attachment factor, high *O*-sulfonation may be an important feature	(Klimyte et al., 2016)
Polyomaviridae		
Human polyomavirus 2	Binds GAGs as attachment factors but not essential for entry	(Cagno et al., 2019)
Merkel cell polyomavirus	Binds to HS and DS as initial attachment factor	(Schowalter et al., 2011)
Bunyaviridae		
Rift Valley Fever Virus	Binds HS as attachment. Infection reduced HS-deficient cells	(de Boer et al., 2012)
Crimean-Congo haemorrhagic fever virus	High HS in sera of infected patients may play a role in haemorrhagic pathophysiology	(Guven et al., 2013)
Hepevirus		
Hepatitis E	Binds HS as an essential attachment factor	(Kalia et al., 2009)
Poxviridae		
Vaccinia Virus	Binds a variety of GAGs, primarily HS. Required for infection	(Lin et al., 2000)
Caliciviridae		
Norovirus genogroup 2	Binds HS on host cell surface - sulfonation very important	(Tamura et al., 2004)
Retroviridae		
Human immunodeficiency virus	Binding HS increases efficiency. Not required for attachment	(Connell and Lortat-Jacob, 2013; Pomin et al., 2017)
Human T-cell leukemia virus type	Binding HS is essential for entry into host cell	(Jones et al., 2006)
Hepadnaviridae		
Hepatitis B	Binding HS as attachment factor essential entry host cell	(Leistner et al., 2008; Lamas Longarela et al., 2013)
Rhabdovirus		
Rabies Virus	Binds HS as an attachment factor but not essential for entry	(Sasaki et al., 2018)
Papillomaviridae		
Human papillomavirus	Binds HS as initial binding receptor which facilitates movement to a specific uptake receptor	(Giroglou et al., 2001)
Bundibugyo ebolavirus	Binds variety GAGs. Sulfonation level affect bind capacity	(Salvador et al., 2013; O’Hearn et al., 2015)

GAG, glycosaminoglycan; HS, heparan sulfate; DS, dermatan sulfate.

The heavily sulfonated chains of cell-surface GAGs present a global negative charge that can interact electrostatically with basic residues of viral capsid proteins or viral surface glycoproteins of enveloped viruses (Cagno et al., 2019). Viruses utilize these interactions to increase their concentration at the cell surface and increase the chances of binding a more specific entry receptor and initiating the infection process (Rusnati et al., 2009). In some cases, GAGs act directly as the primary attachment receptor (**Figure 1B**), such as HSV (O’Donnell and Shukla, 2008). HSV-1 envelope glycoproteins gB and/or gC initiates the viral interaction with HS, followed by the binding of gD to a secondary receptor to initiate membrane fusion with the host cell (O’Donnell and Shukla, 2008). Specific positively charged regions of gC interact with 6-*O*- and 2-*O*-sulfate groups on HS to confer binding (Feyzi et al., 1997). Additionally, a short lysine-rich region of gB which is required for gB-mediated HSV attachment has been identified as the HS binding domain (Laquerre et al., 1998). GAGs also act as mediators for the initial endocytosis of viral particles (**Figure 1B**), which controls the virulency and pathogenicity of infection (Bauer et al., 2021). A sufficient sulfate content of GAGs has been shown to be integral in this process, as several studies have shown that treatment with sulfonation inhibitors, enzymatic removal of sulfate or culturing cell lines in sulfate-deficient conditions reduces infection (Trybala et al., 2000; Mandl et al., 2001; Su et al., 2001; Germi et al., 2002; Tamura et al., 2004).

Due to this role in the initial infection process, GAGs have garnered interest in prophylactic and therapeutic antiviral studies. Treating virus particles with GAGs was shown to inhibit binding of surface glycoproteins to host cell receptors, preventing entry and effectively neutralizing the virus (Leonova and Belikov, 2019). Heparinized blood has also been shown to inhibit binding and entry of pathogens known to interact with host cell GAGs (Aquino and Park, 2016). Additionally, some viruses that do not use GAGs *in vivo* become GAG-dependent after repeated passage in cell culture, resulting in improved viral fitness and out-competing of GAG-independent variants (Cagno et al., 2019). As these viruses can rapidly adapt to utilizing GAGs in cultured cells, similar adaptations have the potential to occur during human infections to promote replication and infection.

### 5.2 Bacteria

This study identified 28 pathogenic bacteria that bind GAGs or utilize ectodomain shedding of GAGs to promote pathogenesis, of which 11 are gram-positive and 17 are gram-negative (**Table 2**). GAGs are involved in adhesion and internalization of bacterial pathogens, including both gram-negative and gram-positive bacteria (Garcia et al., 2016a). HS proteoglycans on the cell surface mediate endocytosis of several HS-binding ligands (**Figure 1B**), although the precise mechanisms leading to ligand internalization are not completely understood (Bartlett and Park, 2011). Certain bacteria have adapted to subvert this mechanism for entry and colonization of host cells. A sufficient degree of sulfonation of these GAGs is required to facilitate this binding, with studies showing that treatment with sulfonation inhibitors or enzymatic removal of sulfate reduces infection (Noel et al., 1994; Rosmarin et al., 2012; Rajas et al., 2017). For example, host cell HS is a receptor for the Group B *Streptococcus* surface protein ACP. ACP-HS binding was shown to facilitate internalization of Group B *Streptococcus* via mechanisms requiring rho GTPase-mediated actin polymerization (Kamhi et al., 2013). Higher degree of polymerization and negative charge are also critical to ACP interactions, as infectivity is markedly decreased in host cells deficient in HS polymerases or *N*-sulfotransferases (Chang et al., 2011).

**Table 2:** Bacterial pathogens that interact with sulfonated GAGs during infection process.

Bacteria	Mechanism	References
*Staphylococcus Aureus*	Binds to HS as a cofactor, promoting adherence. Also induces shedding of heparin-binding EGF which induces mucin overexpression, promoting lung infection by obstructing airflow and inhibiting antibacterial agents	(Liang et al., 1992; Chen et al., 2008)
*Listeria monocytogenes*	Binds to HS promoting adherence and invasion into epithelial cells	(Henry-Stanley et al., 2003)
*Mycobacterium tuberculosis*	Binds HS to facilitate initial attachment and entry into host cell	(Menozzi et al., 2006; Zimmermann et al., 2016)
*Lactobacillus salivarius*	Binds to GAGs as a co-receptor for initial adherence	(Martín et al., 2013)
*Streptococcus pneumoniae*	Stimulates ectodomain shedding of cell surface HS to promote pathogenesis	(Chen et al., 2007)
*Streptococcus pyogenes*	Stimulate ectodomain shedding of DS which bind to and inactivate neutrophil-derived α-defensins, promoting pathogenesis	(Frick et al., 2003)
*Streptococcus agalactiae*	Interacts host cell surface HS to transcytose and facilitate invasive disease	(Baron et al., 2004)
*Enterococcus faecalis*	Stimulate ectodomain shedding of DS which bind to and inactivate neutrophil-derived α-defensins, promoting pathogenesis	(Schmidtchen et al., 2001)
*Bacillius cereus*	Stimulated shedding of cell surface HS from epithelial cells and compromise epithelial barrier integrity, promoting pathogenesis	(Popova et al., 2006)
*Bacillius antracis*	Stimulates shedding of HS ectodomain, increasing barrier permeability and thereby contributing to dissemination of infection, haemorrhages and oedema. Shed ectodomains can also function as paracrine or autocrine effectors	(Popova et al., 2006)
*Streptococcus mutans*	Binds sulfate-containing GAGs in heart tissue	(Choi and Stinson, 1989)
*Chlamydia Trachomatis*	Binds HS as an attachment factor to initiate colonisation. Degree of attachment strongly correlates with degree of sulfation.	(Rosmarin et al., 2012)
*Pseudomonas aeruginosa*	HS is necessary and sufficient to medicate attachment to host cells. Also stimulates ectodomain shedding of DS which bind to and inactivate neutrophil-derived α-defensins and thereby promote pathogenesis neutrophil-derived α-defensins and thereby promote pathogenesis	(Schmidtchen et al., 2001)
*Neisseria gonorrhoeae*	Binds to HS and subsequently facilitates cell entry through HS receptor cytoplasmic domain interactions	(van Putten and Paul, 1995; Freissler et al., 2000)
*Haemophilus influenzae*	Binds HS and DS to facilitate adherence to host cells. Decreased adherence is observed in cells expressing under-sulfonated HS and adherence is inhibited in presence of soluble DS.	(Noel et al., 1994)
*Chlamydia pneumoniae*	Binds HS as an attachment cofactor- enzymatic removal of surface HS from the host cell resulted in a marked reduction infection	(Wuppermann et al., 2001)
*Bordetella pertussis*	Sulfate is released from damaged respiratory epithelial cells which can modulate virulence factor expression in *B. Pertussis*	(Luu et al., 2018)
*Borrelia burgdorferi*	Binds sulfonated-GAGs in initial attachment. GAG is cell-type specific	(Leong et al., 1998)
*Neisseria meningitidis*	Binds HS as an attachment receptor	(Serruto et al., 2010)
*Helicobacter pylori*	Binds HS. Also secretes heparanase which facilitates the colonization in the gastric mucosa	(Dubreuil et al., 2002)
*Orientia tsutsugamsuhi*	Binds HS as initial entry factor	(Kim et al., 2004)
*Porphyromonas gingivalis*	Induces HS shedding, promoting pathogenesis	(Dubreuil et al., 2002; Andrian et al., 2005)
*Yersinia enterocolitica*	Secretes toxic virulence factors that bind HS- sabotages the communication networks of the host cell or even to causes cell death	(Boyd et al., 1998)
*Escherichia coli*	Binds HS as a co-attachment factor, also observed to bind other GAGs	(Rajas et al., 2017)
*Klebsiella pneumoniae*	Binds HS as a co-attachment factor, also observed to bind other GAGs	(Rajas et al., 2017)
*Serratia marcescens*	Binds HS as a co-attachment factor, also observed to bind other GAGs	(Rajas et al., 2017)
*Treponema pallidum*	Binds HS. Sulfonated proteoglycans also accumulate during infection	(Alderete and Baseman, 1989)
*Haemophilus ducreyi*	Binds HS as a co-attachment factor	(Frisk and LagergÅRd, 1998)

GAG, glycosaminoglycan; HS, heparan sulfate; DS, dermatan sulfate.

Additionally, upregulated expression of certain GAGs following tissue injury or epithelial damage is proposed to play a role in increased propensity for bacteria to cause infection in the context of tissue damage and repair (Bartlett and Park, 2011). Studies have shown that the presence of a mixture of GAGs inhibited adhesion to the same extent as when using only HS in gram-positive bacteria. However, the use of a combination of different GAGs significant increased inhibition compared to only HS in gram-negative bacteria, suggesting that HS is the primary GAG used but other GAG species are also involved for these microorganisms (Garcia et al., 2016b).

GAGs are also observed to promote bacterial infection by serving as a soluble inhibitor of innate immunity when released into the extracellular environment via ectodomain shedding (Aquino et al., 2022). Ectodomain shedding via enzymatic cleavage of cell surface GAGs, most commonly the HS proteoglycan sydecan-1, can be induced by certain bacterial pathogens either by hijacking host cell machinery or secreting ectodomain-cleaving enzymes (Bartlett and Park, 2011). Released sydecan-1 ectodomain then binds to and inhibits host immune factors, such as cytokines and antimicrobial peptides, resulting in dysregulation of host immune response and enhancement of pathogenesis (Garcia et al., 2016a).

### 5.3 Parasites and prion

This study identified 7 parasitic organisms and 1 prion particle that interact with GAGs in mammalian infection (**Table 3**). Various parasitic pathogens have been observed to use GAGs as adhesion receptors to attach to host cells (Kamhi et al., 2013). Mast cells, the primary immune cells involved in protecting against parasitic infections, are particularly rich in highly sulfonated GAGs. These GAGs are released during degranulation in response to parasites (Mulloy et al., 2017). Some parasites, much like bacteria, can synthesize or induce shedding of host GAGs to modulate the host immune response and enhance pathogenicity (Kamhi et al., 2013). HS on the surface of erythrocytes has shown to be important, if not essential, for the binding and entry of *Plasmodium falciparum*, however the exact mechanisms are not yet known (Kobayashi et al., 2010).

**Table 3:** Parasites and prion that interact with sulfonated GAGs during infection process.

Organism	Mechanism	References
*Giardia lamblia*	Binds to GAGs, particularly HS, a common GAG in the intestinal tract	(Weiland et al., 2003)
*Leishmania* spp	Binds HS to varying affinities	(Maciej-Hulme et al., 2018)
*Plasmodia* spp.	Binds HS in host cell invasion and motility- migrate through cells expressing low-sulfonated HS, while highly-sulfonated HS facilitates cellular invasion.	(McCormick et al., 1999; Coppi et al., 2007; Kobayashi et al., 2010)
*Toxoplasma gondii*	Binds HS as initial attachment factor	(Jacquet et al., 2001; Bishop et al., 2005; Bannai et al., 2008)
*Trypanosoma cruzi*	Binds HS as an attachment and entry factor in cardiomyocytes	(Lima et al., 2002; de Oliveira et al., 2008)
*Encephalitozoon* spp.	Spore adheres to host cell surface GAGs (HS and CS) in vitro- modulates infection process	(Southern et al., 2007)
*Fasciola hepatica*	DS and HS are involved in tissue invasion processes	(Beckham et al., 2006)
Prion	Binds HS for attachment and entry to host cells- may also play role in intracellular trafficking	(Horonchik et al., 2005; Taylor et al., 2009)

GAG, glycosaminoglycan; HS, heparan sulfate; CS, chondroitin sulfate.

Prion diseases are untreatable and fatal neurodegenerative diseases that result from conversion of a normal cell surface protein into a pathological conformation that is transmissible (Westergard et al., 2007). Enzymatic removal of surface HS, prevention of sulfonation with chlorate or presence of competing sulfonated glycans prevent binding and internalization of infectious prion rods, indicating cell surface HS is required for prion infection (Horonchik et al., 2005). HS is also proposed to play a role in the intracellular trafficking of pathogenic prions (Horonchik et al., 2005).

## 6 Conclusion

In conclusion, GAGs are involved in the infection process of numerous pathogens and sufficient sulfate content is needed to facilitate these interactions. Circulating sulfate levels are decreased or increased by several factors, leading to altered sulfate content of GAGs which in turn is proposed to subsequently compromise or enhance infection of GAG-binding pathogens. Therapeutic approaches for targeting GAG-pathogen interactions have the potential to reduce pathogen infection. Initial results from *in vitro* and cell culture studies have increased clinical interest for future prophylactic and therapeutic antipathogen treatments.

Recent studies have focused predominantly on certain pathogens that are known to interact with GAGs. This review brings together all known human pathogens that are known to interact with GAGs in infection. In total 59 viruses, 28 bacteria, 7 parasites and 1 prion were identified, showing that the use of GAGs is a highly conserved feature (**Figure 1C**). These findings provide a resource for future studies and highlight the need for further studies to investigate the consequences of high or low sulfatemia on pathogen infection.
